# Melatonin as the Missing Link Between Sleep Deprivation and Immune Dysregulation: A Narrative Review

**DOI:** 10.3390/ijms26146731

**Published:** 2025-07-14

**Authors:** Ida Szataniak, Kacper Packi

**Affiliations:** 1 Wladyslaw Bieganski Collegium Medicum, Jan Dlugosz University in Czestochowa, 42-200 Częstochowa, Poland; idaszataniak@gmail.com; 2 Department of Nucleic Acid Biochemistry, Medical University of Lodz, 92-213 Lodz, Poland; 3 AllerGen Center of Personalized Medicine, 97-300 Piotrkow Trybunalski, Poland

**Keywords:** sleep deprivation, melatonin, immune system, circadian rhythm, inflammation, immunomodulation

## Abstract

Sleep deprivation impairs immune function, and melatonin has emerged as a key mediator in this process. This narrative review analyzes 50 studies published between 2000 and 2025 to determine the extent to which reduced melatonin synthesis contributes to immune dysregulation. Consistent sleep loss lowers melatonin levels, which correlates with elevated proinflammatory cytokines (e.g., IL-6 and TNF-α), increased oxidative stress, and reduced immune cell activity, including that of natural killer (NK) cells and CD4+ lymphocytes. Melatonin regulates immune pathways, including NF-κB signaling. It also supports mitochondrial health and helps maintain gut barrier integrity. These effects are particularly relevant in vulnerable populations, including older adults and shift workers. Experimental findings also highlight melatonin’s therapeutic potential in infections like SARS-CoV-2, where it modulates inflammatory responses and viral entry mechanisms. Despite the heterogeneity of study methodologies, a consistent correlation emerges between circadian disruption, melatonin suppression, and immune imbalance. These findings underscore melatonin’s dual role as a chronobiotic and immunomodulator. Addressing sleep loss and considering melatonin-based interventions may help restore immune homeostasis. More clinical trials are needed to determine the best dosing, long-term efficacy, and population-specific strategies for supplementation. Promoting healthy sleep is crucial for preventing chronic inflammation and diseases associated with immune dysfunction.

## 1. Introduction

Sleep is a critical regulator of circadian rhythms and systemic physiological homeostasis [1], and its disruption leads to multiple downstream effects on metabolic and immune functions [2]. One of the central mediators of these effects is melatonin, a hormone secreted primarily during nocturnal hours that plays a pivotal role in synchronizing the circadian clock with environmental light–dark cycles [3]. However, factors such as chronic sleep deficits occurring in insomnia, alcoholism, stress, and aging have been demonstrated to significantly suppress melatonin secretion [4]. Moreover, melatonin has been shown to exhibit anti-inflammatory properties, a factor that may be significant in light of the evidence of inflammation observed in some individuals with autism spectrum disorders (ASD) [5]. Melatonin interacts with receptors found in various immune cells and organs, highlighting its key role in immune regulation [6]. Preclinical studies have shown that melatonin has been shown to act as a potent antioxidant, protecting immune cells from oxidative stress [7]. Its immunoregulatory properties extend to both innate and adaptive immunity, including the enhanced activity of natural killer (NK) cells and regulation of CD4+ lymphocytes [8]. Preclinical studies suggest that melatonin exerts immunoregulatory effects on both innate and adaptive immunity, including the enhanced activity of natural killer (NK) cells and modulation of CD4+ lymphocyte function [8].

Non-clinical studies have indicated that sleep deprivation leads to an increase in the secretion of pro-inflammatory cytokines, such as IL-6 and TNF-α [9]. These cytokines play a crucial role in regulating sleep under physiological conditions [9]. However, their chronic elevation has been associated with fatigue and immunosuppression [10]. Studies in humans have shown that prolonged sleep restriction has been demonstrated to alter HPA axis activity, resulting in elevated cortisol levels, which may influence immune modulation [11].

Our objective in this narrative review is to examine the effects of sleep deprivation on melatonin synthesis and its impact on immune system function. As individuals age, these issues become increasingly evident. Aging is associated with reduced melatonin production and an increased prevalence of insomnia [12], which contributes to immune dysregulation and increased susceptibility to infection in older adults [13,14]. Experimental and clinical studies suggest that nutritional interventions, including the consumption of tryptophan-enriched foods, have been demonstrated to support the synthesis of melatonin within the body [15]. These strategies may counteract the immunosuppressive effects of chronic sleep loss [15]. Preclinical studies in animal models have demonstrated that sleep deprivation alters the composition of the gut microbiota, leading to a decrease in short-chain fatty acids (SCFAs). [16] These SCFAs have been shown to play a crucial role in microglial regulation and anti-inflammatory signaling [16]. Reduced levels of short-chain fatty acids (SCFAs) have been observed to potentially contribute to neuroinflammation and cognitive deficits that manifest subsequent to sleep deprivation [16]. Moreover, preclinical data from rodent models indicate that sleep deprivation has been demonstrated to affect circadian gene expression, which plays a critical role in memory and immune function by regulating B cell maturation and cytokine secretion of natural killer (NK) cells [17,18]. Melatonin, a hormone that is suppressed during sleep loss, has been shown to synchronize circadian and immune rhythms [18]. Additionally, it has been demonstrated to reduce the risk of viral infections, such as SARS-CoV-2, by maintaining the nighttime immune defense [19]. Its immunomodulatory effects have also been demonstrated in vulnerable populations, including children with Down syndrome [20]. Furthermore, in animal models, research has demonstrated that chronic sleep loss and related stress activate the HPA axis, leading to persistent glucocorticoid release, which suppresses both cellular and humoral immune responses [21]. The present findings underscore the intricate interplay among sleep, circadian regulation, and immune function. They further suggest that melatonin may function as a promising therapeutic modulator in conditions associated with sleep loss [22,23].

## 2. Results

### 2.1. Study Selection

A comprehensive narrative review was conducted to investigate the potential causal relationship between sleep deprivation, melatonin synthesis, and immune system function. A total of 20 articles were excluded based on predefined inclusion criteria, resulting in a final selection of 50 studies. These studies formed the basis of the review and were analyzed in detail, with additional contextual discussion provided where relevant.

A narrative review was conducted to explore the potential causal relationship between sleep deprivation, melatonin synthesis, and immune system function. The initial literature search yielded a large number of articles. After screening the titles and abstracts, 70 potentially relevant studies were selected for a full-text review.

Twenty articles were subsequently excluded according to predefined inclusion and exclusion criteria that considered factors such as language, publication type, relevance to the review topic, and methodological quality. Although this review did not use a formal quality assessment tool, studies were excluded due to low perceived methodological rigor if they met one or more of the following general conditions:

1. Insufficient clarity regarding the study population or sampling method;
2. Absence of a control or comparison group;
3. Lack of clearly defined outcomes;
4. Very small sample size with no justification of power.

These exclusion criteria were applied to focus the review on studies with greater internal validity and scientific transparency. Ultimately, the final analysis included 50 studies, which were reviewed in depth with additional contextual commentary where appropriate.

### 2.2. Study Characteristic

The included studies exhibited considerable heterogeneity, encompassing case studies, clinical trials, meta-analyses, and observational research. The majority of the selected articles, consistent with the objective of this review, investigated melatonin as an immunomodulator in conditions associated with sleep deprivation. The findings from these studies indicated, among other outcomes, that melatonin mitigated inflammatory markers such as TNF-alpha and IL-6. A number of the reviewed studies utilized experimental models, applying sleep deprivation protocols to assess the impact of melatonin on immune responses.

### 2.3. Results of Individual Studies

Reduced melatonin production and increased cortisol levels in elderly individuals may contribute to impaired immune function [24]. Similarly, people suffering from insomnia exhibit lower melatonin levels and impaired immune responses [10]. Furthermore, people who work at night often have their melatonin levels suppressed due to constant exposure to light during that time of day [25]. This has been associated with higher rates of respiratory infections and cardiovascular issues [25]. Similarly, individuals with autism spectrum disorders tend to have lower melatonin levels, which correlate with immune irregularities, including reduced natural killer (NK) cell function and increased oxidative stress [5]. Additionally, preclinical studies, including animal models and in vitro experiments using immune cells, have demonstrated that melatonin plays a key role in supporting mitochondrial function [26]. It helps maintain ATP synthesis and prevents immune cell death during periods of immune stress [26]. These effects are essential for maintaining energy balance in activated immune cells, particularly under inflammatory or infectious conditions. It is also worth noting that in patients with gastrointestinal conditions, melatonin has demonstrated anti-inflammatory properties in gut tissues and the ability to improve mucosal immunity [27]. Furthermore, studies investigating melatonin’s role in gastrointestinal and metabolic health support its protective function in maintaining mucosal immunity. [27] Melatonin supplementation has been suggested to restore normal melatonin levels and support antioxidant and immunomodulatory functions, as demonstrated in various experimental models [28]. Likewise, dietary supplementation with melatonin or its precursor, tryptophan, has been shown to restore sleep quality and enhance immune cell functionality [29]. Nutritional studies also show that combining melatonin-rich foods with proper sleep hygiene contributes to restoring circadian hormonal rhythms and enhancing immunological resiliency [30]. Beyond these effects, melatonin has demonstrated immunomodulatory effects in clinical settings, including as an adjuvant during chemotherapy, where it enhances natural killer (NK) cell activity and reduces inflammation [31]. In addition, melatonin interacts with receptors found in various immune cells and organs, highlighting its key role in immune regulation [6]. Table 1 highlights the key clinical conditions and vulnerable populations in which melatonin has demonstrated significant immunomodulatory effects. These examples illustrate its broad therapeutic potential across diverse immunological contexts.

The synthesis of the findings across multiple studies reveals a strong and consistent association between sleep deprivation and reduced melatonin synthesis. These disruptions result in circadian misalignment and hormonal imbalances, including elevated cortisol levels [24,27]. Observational and clinical data indicate that irregular sleep schedules are linked to reduced melatonin production, which may impair immune function [23]. Clinical studies have shown that melatonin production, which is governed by the suprachiasmatic nucleus (SCN) and regulated by exposure to darkness, declines markedly when normal sleep patterns are disturbed [33].

A number of studies have reported that sleep deprivation increases circulating levels of cytokines linked to chronic inflammation and heightened susceptibility to infections [34]. These inflammatory markers, in turn, have been shown to impair sleep quality, thereby reinforcing a cycle of immune dysregulation [10]. The relationship between decreased melatonin levels and increased concentrations of proinflammatory cytokines, including IL-6, IL-1β, and TNF-α, has been demonstrated to be consistent [27]. This assertion is further substantiated by the experimental data, which demonstrates that melatonin exerts an upregulatory effect on protective cytokines such as IL-2 and IFN-γ [6,10]. Furthermore, the absence of adequate sleep has been shown to induce alterations in T-cell function, to diminish NK cell activity, and to trigger the hypothalamic–pituitary–adrenal (HPA) axis, thereby contributing to a state of immunosuppression [10]. Melatonin has been demonstrated to inhibit the activity of macrophages, resulting in diminished levels of IL-1β, TNF-α, and nitric oxide, as well as decreased oxidative stress [32]. The efficacy of these effects has been demonstrated in both in vivo and in vitro models [32]. Melatonin has also shown immunomodulatory effects in at-risk groups, such as children with Down syndrome [20]. The combined effects of melatonin deficiency and sleep deprivation on the key immune parameters in both animal and human models are summarized in Table 2.

**Figure 1:**
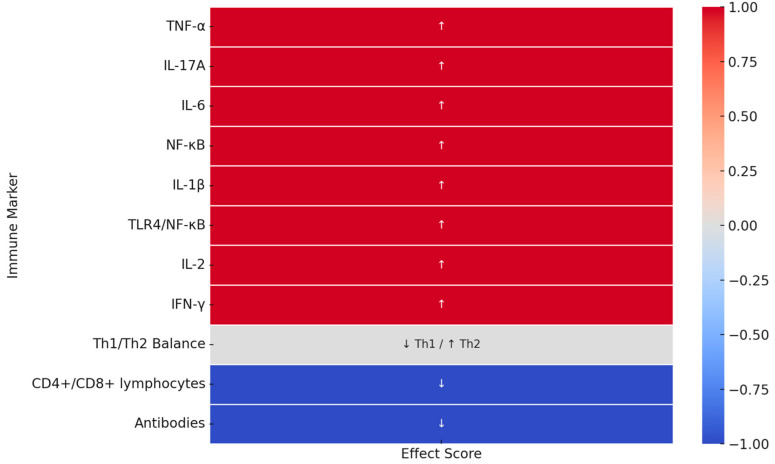
Symbolic representation of directional changes in immune markers under conditions of sleep deprivation and reduced melatonin. Values indicate qualitative trends only, based on the literature: 1 = increase (↑), −1 = decrease (↓), 0 = mixed effect (↓ Th1/↑ Th2).

To facilitate the interpretation of the immune changes associated with sleep deprivation and reduced melatonin levels, the immunological effects presented in Table 2 were recoded using a symbolic scoring system and visualized in Figure 1. This qualitative approach was chosen due to the absence of standardized numerical values in the referenced studies. The scores are not quantitative; they are purely illustrative. A value of 1 (red) indicates the increased expression or activity of a given immune marker, −1 (blue) indicates decreased expression or function, and 0 (gray) indicates a mixed effect, such as an immunological shift (e.g., decreased Th1/increased Th2). This simplified coding system provides a summary of the direction of immune modulation under conditions of sleep deprivation and melatonin deficiency in both animal and human models.

**Figure 2:**
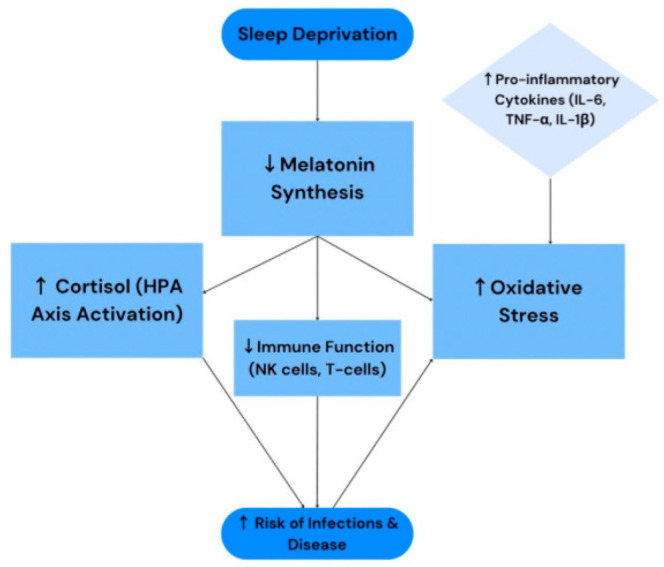
Mechanistic synthesis of relationships between sleep deprivation, melatonin, and immune function. The symbol ↑ denotes an increase, and ↓ denotes a decrease.

Overall, the evidence supports the conclusion that melatonin serves as a critical link between circadian regulation and immune function. Its suppression as a result of sleep loss represents a plausible mechanism underlying the increased vulnerability to inflammation and disease. These effects are consistently observed across different populations and biological systems, lending robust external validity to the proposed model. Melatonin, therefore, emerges as a central factor, acting both as a chronobiotic hormone and a key immunomodulator [29]. The relationships between sleep deprivation, melatonin levels, and immune function are illustrated in Figure 2. 

Consequently, any disruption to circadian timing, such as that resulting from insufficient sleep, can significantly impair this process. It is noteworthy that melatonin not only functions as a regulator of the circadian clock but also as a potent immunomodulatory hormone [6,37]. In animal models and in vitro studies, melatonin has been shown to reduce oxidative stress by scavenging free radicals and upregulating antioxidant defense mechanisms [38].

A multitude of studies provide substantiation for the notion that sleep deprivation shifts the immune balance toward a more proinflammatory state [27]. Preclinical data suggest that lower melatonin levels may disinhibit the hypothalamic–pituitary–adrenal (HPA) axis, resulting in increased cortisol secretion [21].

Elevated cortisol levels have been demonstrated to impair immune function by reducing lymphocyte activity and promoting systemic inflammation [4]. These effects appear to be particularly salient in aging populations. A decline in melatonin production with age has been demonstrated to coincide with an elevated risk of infection, as evidenced by numerous studies [12]. It has been demonstrated that individuals have exhibited improvements in immune markers following melatonin supplementation, highlighting its therapeutic potential [39]. Furthermore, research indicates that intestinal and metabolic disorders associated with circadian misalignment are exacerbated by melatonin deficiency [27,40], suggesting the presence of broader systemic consequences.

### 2.4. Certainty of Evidence

The findings of this review establish a significant relation between sleep deprivation and melatonin suppression, resulting in weakened immune system function. The conclusions drawn from the available studies also suggest a clear mechanism through which sleep deprivation affects immune function. However, certain inconsistencies in the data may marginally lower the confidence in the evidence in this context. Although a strong association has been demonstrated, uncertainty remains due to methodological differences in melatonin measurement, which may affect the reliability of findings in certain subgroups.

This review establishes a significant relationship between sleep deprivation, melatonin suppression, and dysregulated immune function. Multiple lines of evidence support a biologically plausible mechanism linking circadian misalignment, HPA axis activation, and proinflammatory cytokine release to reduced melatonin availability.

However, it is important to recognize that much of the data originates from preclinical studies, including animal models and in vitro experiments. For instance, animal studies have demonstrated that melatonin significantly reduces pro-inflammatory cytokine levels, modulates oxidative stress, and supports mitochondrial integrity. Similarly, preclinical data suggest that melatonin administration can enhance natural killer (NK) cell activity and restore T-cell balance under sleep-deprived conditions.

While these findings provide a strong mechanistic rationale, the clinical evidence is more limited and inconsistent in some cases. Clinical studies have shown the potential benefits of melatonin supplementation, particularly for elderly populations, individuals with sleep disorders, and patients undergoing chemotherapy. However, the results vary due to differences in study design, outcome measures, and sample sizes. Some of these trials lacked rigorous controls or used heterogeneous populations, which complicates generalizability.

Furthermore, certain associations reported in the literature, such as those linking melatonin to cytokine regulation, are based on indirect or correlative data, especially when extrapolated from different pathological or physiological contexts. While the reviewed studies collectively suggest a strong immunomodulatory role for melatonin, the degree to which these effects translate into clinical benefit is uncertain.

Most of the above findings are based on preclinical models, and their clinical relevance has yet to be fully confirmed. High-quality randomized controlled trials are needed to validate these mechanisms in humans and clarify melatonin’s therapeutic potential in immune-related conditions exacerbated by sleep deprivation.

At the same time, we acknowledge that some of the associations discussed, particularly those related to cytokine modulation, are supported by indirect or preclinical evidence, and certain mechanistic pathways have not yet been definitively proven in humans. Nonetheless, given the consistency of these findings across different models and their alignment with known biological pathways, we believe their inclusion is justified as a basis for further clinical investigation.

## 3. Discussion

### 3.1. Interpretation of the Results in the Context of the Other Evidence

The lack of randomized clinical trials in humans prevents a conclusive assessment of the immunomodulatory efficacy of melatonin, as well as the determination of its optimal dosage and long-term safety [41]. Until these data are completed, melatonin remains a promising but not fully validated therapeutic tool. In the context of assessing stress responses, a promising direction may be the simultaneous measurement of IL-1β, cortisol, and melatonin. IL-1β may serve as an accurate physiological marker, particularly in situations perceived as aggressive, which may allow better monitoring of the immunological effects of stress in the future [42]. Chronic sleep restriction results in a persistent low-grade inflammatory state that increases the risk of developing chronic diseases such as atherosclerosis, type 2 diabetes, or autoimmune disorders [43]. Therefore, routine evaluation and treatment of sleep disorders should become an integral part of the prevention and treatment of inflammatory conditions.

As the main signal for the synchronization of peripheral clocks, melatonin plays a key role in maintaining the rhythmic activity of immune cells. Its deficiency due to sleep deprivation leads to desynchronization of molecular rhythms in leukocytes and disruption of their effector and regulatory functions. This can lead to chronic immune dysregulation and increased susceptibility to infection, implicating melatonin as a potential mechanistic link between sleep disturbance and immune dysregulation [44]. This demonstrates the need to implement sleep protection programs among shift workers.

For instance, melatonin also acts through the RORA and TLR4/TRIF pathways, making it a particularly interesting therapeutic target in the context of immune disorders associated with sleep deprivation [45]. Its supplementation may have future applications in the prevention and treatment of immune dysregulation associated with circadian rhythm disruption [45]. Preclinical data suggest that melatonin acts through the RORA and TLR4/TRIF pathways, making it a particularly interesting therapeutic target in the context of immune disorders associated with sleep deprivation [45]. These findings, observed primarily in animal models and cell-based studies, indicate that melatonin supplementation may have potential future applications in the prevention and treatment of immune dysregulation related to circadian rhythm disruption [45].

Furthermore, genetic links between sleep and the immune system, including the presence of genes such as IL-1β and TNF in sleep-regulating loci, provide strong evidence for a molecular coupling between these two systems. These findings pave the way for developing targeted therapies that account for the shared regulation of sleep and immunity [46]. Genetic studies in humans have revealed links between sleep and the immune system, including the localization of immune-related genes such as *IL-1β* and *TNF* within sleep-regulating loci. These clinical findings provide compelling evidence for a molecular coupling between sleep and immunity [46]. Such insights may help inform the development of targeted therapies that address the shared regulation of these two systems [46].

Sleep deprivation damages the intestinal barrier, disrupts the microbiota, and weakens cellular junctions. A significant decrease in melatonin levels suggests that its deficiency may be a key factor in these changes. Melatonin supplementation restores intestinal integrity, suggesting its potential role in the treatment of mucosal barrier dysfunction in individuals with chronic sleep deprivation [47]. In animal models, sleep deprivation damages the intestinal barrier, disrupts the microbiota, and weakens cellular junctions. A significant decrease in melatonin levels in sleep-deprived mice suggests that its deficiency may be a key factor in these changes. Melatonin supplementation restored intestinal integrity in mice, suggesting its potential therapeutic role in preventing mucosal barrier dysfunction under conditions of chronic sleep deprivation [47].

Supplementing with melatonin could be a potential therapeutic approach to restore intestinal integrity and treat mucosal barrier dysfunction in individuals with chronic sleep deprivation.

Viral infections alter sleep by reducing REM sleep and prolonging NREM sleep. This may represent an adaptive mechanism to support the immune response, suggesting that sleep structure is actively regulated during infection. [48]. Evidence from human studies suggests that viral infections reduce REM sleep and increase NREM sleep duration. This shift in sleep structure is hypothesized to be an adaptive response facilitating immune function during infection [48].

Understanding how viral infections affect sleep patterns could lead to strategies that optimize sleep during illness, enhancing immune function. Clinically, interventions aimed at improving sleep quality, particularly by restoring REM sleep, may support the body’s immune response and improve recovery during viral infections.

The postprandial increase in melatonin levels observed in birds suggests the existence of an alternative, food-dependent mechanism for synchronizing biological rhythms. If a similar mechanism operates in humans, gut-derived melatonin may become a new therapeutic target for the treatment of circadian, metabolic, and inflammatory disorders [49,50]. In the elderly, decreased melatonin and serotonin levels can lead to sleep disturbances and immune system aging. Clinically, tryptophan supplementation may offer a potential intervention to restore these neurotransmitters, improve sleep quality, and help mitigate age-related immunological decline [50].

These findings highlight the need for targeted therapeutic approaches, such as melatonin supplementation, to restore these systems, particularly in vulnerable populations such as shift workers and the elderly. While the lack of randomized clinical trials in humans prevents definitive conclusions, the promising effects of melatonin on immune function, sleep quality, and gut health open opportunities for further research. In addition, understanding the interplay between sleep and immunity may lead to the development of new, targeted therapies that address the joint regulation of these systems, ultimately improving overall health and reducing the risk of chronic disease and infection.

### 3.2. Limitations of the Evidence Included in the Review

One of the main limitations which we encountered was the variability in research methodologies. The sleep deprivation protocols differed across the studies. These differences included the duration and intensity of sleep deprivation, as well as the characteristics of the participants. Another limitation was the significant predominance of studies examining short-term exposure to sleep deprivation. Long-term studies are scarce, which limits our understanding of chronic effects. While acute sleep deprivation provides valuable insights, it may not fully reflect the biological consequences of prolonged sleep disruption.

Additionally, many of the studies used animal models that do not fully replicate human physiology. Lastly, much of the existing evidence is correlational, which limits our ability to draw definitive causal inferences about the relationship between sleep deprivation, melatonin suppression, and immune function.

### 3.3. Limitations of the Review Process Used

Despite the use of predefined inclusion and exclusion criteria, the review process had limitations. One significant concern was publication bias, whereby studies with statistically significant or positive results were more likely to be published and retrieved in searches, while studies with null or negative results may be underrepresented. This could affect the balance of evidence considered in the review. Furthermore, the review relied exclusively on secondary data; therefore, no direct experimental verification of the causal relationships between sleep deprivation, melatonin suppression, and immune dysfunction was performed. While many of the included studies suggest robust associations, a large proportion of this evidence is correlational, which limits the ability to draw firm causal conclusions. Another important limitation involves the subjective elements in the selection of the studies. Although the initial screening and eligibility process was based on predefined criteria (e.g., language, publication type, relevance, and methodological quality), the final selection of studies was also influenced by how well they aligned with the review’s central theme and objectives. Consequently, relevant studies that did not appear to fit the narrative focus may have been unintentionally excluded, introducing an element of selection bias. Additionally, although methodological quality was considered during the screening process, a formal quality assessment was not used. Studies were excluded due to low perceived methodological rigor if they met one or more of the following conditions: insufficient clarity regarding the study population or sampling method, absence of a control or comparison group, lack of clearly defined outcomes, or very small sample size without power justification.

These criteria were applied to ensure the internal validity and scientific transparency of the included studies. However, the lack of standardized scoring or independent validation introduces subjectivity into quality judgments. In summary, although the review aimed to synthesize the most relevant and credible available evidence, the narrative approach and reliance on potentially selective secondary data must be acknowledged as inherent limitations.

### 3.4. Implications for Practice, Policy, and Future Research

Taken together, this review demonstrates that melatonin suppression is a central mechanism through which sleep deprivation leads to immune dysfunction. The consistency of these effects across diverse populations and clinical contexts lends strong support to the proposed biological model. Despite the compelling evidence linking melatonin suppression to immune dysregulation, several knowledge gaps and potential sources of controversy remain. A major limitation of the current research is the heterogeneity of the methodologies used to measure melatonin levels. Differences include sampling times, biofluids analyzed (saliva, plasma, and urine), and assay sensitivity. This variability complicates direct comparisons across the studies and may introduce bias, particularly in subgroup analyses. Furthermore, the extent to which these findings will translate into clinical improvements, especially in complex conditions like those associated with SARS-CoV-2, remains to be seen and requires further validation in large-scale, randomized controlled trials.

Another unresolved issue is the optimal dosing and timing of melatonin supplementation, which may differ depending on chronotype, age, comorbidities, and baseline circadian disruption. Additionally, there has been a degree of controversy surrounding the potential consequences of exogenous melatonin administration for the desensitization of endogenous production over time or its interference with natural circadian adaptation mechanisms. Future studies should aim to standardize the melatonin measurement protocols, investigate the long-term safety and efficacy of supplementation, and explore personalized treatment strategies that account for interindividual variability. These efforts would clarify melatonin’s therapeutic potential and inform evidence-based clinical recommendations. The results of this review have significant implications for clinical practice and public health policy. Greater emphasis must be placed on promoting healthy sleep habits. Sleep should be regarded as a critical preventive strategy to reduce the incidence of diseases. It is crucial to emphasize the importance of sleep hygiene. Promoting adherence to a consistent sleep schedule and minimizing exposure to blue light should be widely encouraged. It is essential to develop guidelines for professionals at risk of sleep deprivation, ensuring appropriate rest periods to prevent the long-term consequences of insufficient sleep. In the early stages, sleep deprivation weakens the immune system, and in later stages, it can contribute to the development of serious diseases, such as cancers, which pose a direct threat to life. Further research should be conducted to identify a specific combination of immunological markers that could serve as a standard for assessing the role of sleep deprivation in impairing the body’s defense mechanisms. Additionally, further studies are needed to determine the optimal dosage of melatonin for treating immune dysfunction and to evaluate the long-term effects of this therapy.

Although this review discusses the promising effects of melatonin, it is important to recognize that long-term use, especially at pharmacological doses, raises several concerns. Chronic supplementation may desensitize melatonin receptors or downregulate endogenous production, which could impair the natural circadian rhythm over time. Furthermore, adverse effects, such as daytime drowsiness, hormonal disruption, and drug interactions, have been reported, particularly in vulnerable populations, such as the elderly or individuals with comorbidities.

Another critical consideration is the distinction between physiological doses (typically 0.1–0.5 mg, which mimic natural nocturnal peaks) and pharmacological doses (often ≥3 mg), as these doses may have different biological effects and safety profiles. While low doses may support circadian alignment, higher doses can act more like a drug, modulating immune responses and oxidative stress in ways that are not yet fully understood.

Future studies should address these concerns by investigating the long-term safety of melatonin, particularly with chronic administration, and by comparing physiological versus pharmacological dosing strategies. A more nuanced understanding of the dose–response relationship and how it interacts with age, chronotype, and baseline melatonin levels is essential for informing the safe and effective clinical use of melatonin.

## 4. Materials and Methods

This narrative review examines the impact of sleep deprivation on melatonin synthesis and its effects on immune system function. We included clinical studies, meta-analyses, randomized controlled trials, and relevant animal studies, particularly murine models. Studies not directly related to the review topic, as well as those published in languages other than English or before 2000, were excluded.

We conducted the literature search in April 2025 using PubMed and Google Scholar. A consistent search strategy was applied to both databases using the following keywords: “sleep deprivation,” “insufficient sleep,” “melatonin synthesis,” “pineal gland,” “melatonin,” “immune system,” “immune function,” “cytokines,” “inflammation,” and “anti-inflammatory.” Only studies published between 2000 and 2025 involving human participants or animal models were considered.

**Figure 3:**
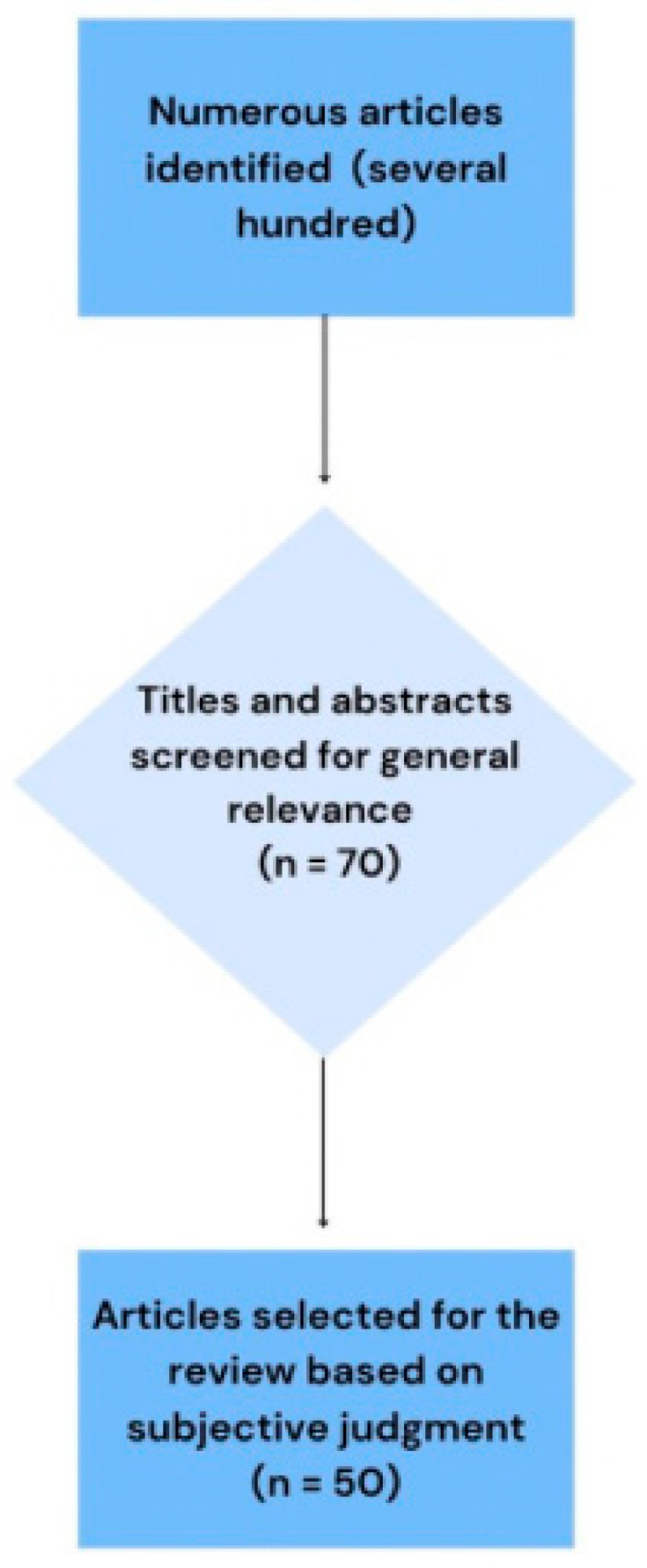
Simplified PRISMA-like flow diagram presenting the study selection process for this narrative review. Articles were identified through database searches, screened based on titles and abstracts, and selected for inclusion based on thematic relevance and expert assessment. Numbers are approximate, reflecting the narrative and integrative approach of this review.

An initial pool of 70 studies was selected based on titles and abstracts. After a full-text review, 20 studies were excluded according to the predefined inclusion and exclusion criteria addressing language, relevance, publication type, and methodological quality. The process illustrating our publication selection is presented in Figure 3, a simplified PRISMA-like flow diagram.

Although this was a narrative (non-systematic) review and no formal scoring system was used, the methodological quality of the studies was assessed based on common scientific standards. Studies were excluded due to low methodological quality if they met one or more of the following conditions:

1. Insufficient clarity regarding the study population or sampling method;
2. Absence of a control or comparison group;
3. Lack of clearly defined outcomes;
4. Very small sample size with no justification of power.

After applying these criteria, 50 studies were included in the final analysis based on the authors’ subjective judgment of the scientific relevance and clarity of each study. The included studies were categorized according to the duration of sleep deprivation, short-term versus chronic, and were analyzed to extract data on serum melatonin levels, lymphocyte subpopulations, cytokine profiles, oxidative stress biomarkers, and gene expression changes related to immune function.

Due to the heterogeneity of the study designs, populations, and outcomes, the results are presented as a narrative synthesis. The key findings were interpreted and summarized through qualitative analysis, allowing expert-driven integration of the available evidence.

## 5. Conclusions

Our review of the most recent studies corroborates the assertion that sleep deprivation impairs immune system function, thereby increasing the susceptibility to infections and inflammatory responses. The research that we have compiled emphasizes the crucial role of sufficient sleep in maintaining physiological homeostasis. Interventions, including public health campaigns, revisions of workplace policies, and public health initiatives, could play a critical role in enhancing population health outcomes. The issue of sleep deprivation and its associated consequences should be incorporated into preventive healthcare strategies, thereby contributing to the prevention of a wide range of diseases.

Looking ahead, several important directions for future research and clinical applications emerge. First, there is a growing need for large-scale, longitudinal, and interventional studies to better understand the long-term impact of melatonin supplementation on immune function and overall health. Randomized controlled trials should particularly assess the safety, efficacy, and optimal dosing strategies for melatonin use across different populations, including older adults, shift workers, and individuals with chronic inflammatory conditions. Personalized approaches that consider chronotype, baseline melatonin levels, and comorbidities may be especially valuable in maximizing the therapeutic benefits while minimizing the potential risks, such as receptor desensitization or hormonal imbalance.

Additionally, developing standardized protocols to measure melatonin concentrations and immune biomarkers, such as IL-1β, TNF-α, IFN-γ, and cortisol, is essential for improving the comparability across studies and defining clinically meaningful thresholds. Integrating sleep-focused assessments into routine healthcare practice could help identify at-risk individuals earlier and enable timely intervention.

Moreover, future research should explore melatonin’s emerging role within the gut–brain–immune axis. The interplay between circadian disruption, gut microbiota alterations, and systemic inflammation suggests that combined interventions involving melatonin, dietary modulation, and pre/probiotics may offer promising therapeutic strategies. This is particularly relevant given melatonin’s demonstrated ability to restore intestinal barrier function and reduce neuroinflammation.

Finally, public health strategies should prioritize policies informed by chronobiology. Promoting sleep hygiene, regulating light exposure, and restructuring shift schedules could help protect circadian integrity and immune resilience. As our understanding of the bidirectional relationship between sleep and immune health improves, melatonin will likely become an important target for preventive and therapeutic interventions.

In conclusion, although the evidence is not yet definitive, melatonin appears to be a key mechanistic link between sleep and immune regulation. Its potential as a chronobiotic and immunomodulatory agent merits further exploration in research and clinical practice.

## Figures and Tables

**Table 1:** Immunomodulatory effects of melatonin across clinical conditions and vulnerable populations.

Clinical Condition/Population	Observation Regarding Melatonin	Effect on the Immune System	Type of Evidence	References
SARS-CoV-2 Infection	Melatonin acts as an adjuvant; reduces cytokine storm	Immunomodulatory, antioxidant, and anti-inflammatory actions; inhibition of Mpro and CD147-mediated viral entry.	Summarizes both clinical and experimental evidence	[19,29]
Down Syndrome (DS)	Melatonin decreases the expression of CD11b, TLR4, MyD88, and NLRP3	Suppression of inflammatory signaling; cytokine modulation.	Experimental ex vivo study	[20]
ASD (Autism Spectrum Disorder)	Reduced melatonin levels in children with ASD	Increased inflammatory markers; potential anti-inflammatory effect of melatonin.	Summarizes both clinical and experimental evidence	[5]
Irritable Bowel Syndrome (IBS)	Disruption of endogenous melatonin levels; improvement with supplementation	Antinociceptive and anti-inflammatory effects; regulation of gastrointestinal motility.	Summarizes both clinical and experimental evidence	[32]
Obesity	Low melatonin levels associated with inflammation	Modulation of innate and adaptive immunity; reduced infection susceptibility.	Summarizes both clinical and experimental evidence	[31]

**Table 2:** Impact of sleep deprivation and melatonin deficiency on immune markers in animal and human models.

Immunological Marker	Effect of Low Melatonin Levels/Sleep Deprivation	Type of Evidence	References
TNF-α	Elevated TNF-α expression in adipose and brain tissue, worsening inflammation.	Preclinical	[10]
IL-17A	Sustained increase even after REM deprivation ends (7 days post-experiment), suggesting long-term proinflammatory effects.	Preclinical	[10]
Th1/Th2 Balance; Cortisol (HPA axis)	Sleep deprivation enhances HPA axis activity and cortisol levels, exerting immunosuppressive effects and shifting immunity from Th1 to Th2.	Clinical and Preclinical	[10,35,36]
CD4+ and CD8+ lymphocytes	Decrease in CD3+, CD4+, and CD8+ cell count in insomnia, secondary to HPA axis and cortisol effects.	Clinical	[10,35]
Antibodies (Humoral Response)	Individuals sleeping < 7 h exhibit significantly weaker vaccine responses.	Clinical and Preclinical	[10,36]
IL-6	Increased IL-6 levels as a result of sleep deprivation, leading to an inflammatory state.	Preclinical	[16]
NF-κB	Activation of transcription factor promoting proinflammatory cytokine expression.	Preclinical	[16]
IL-1β	Increased expression in response to REM deprivation, persisting for several days post-deprivation.	Preclinical	[16]
TLR4/NF-κB activation	Activation of the signaling pathway associated with microbiota and neuroinflammation following microbiota transplantation.	Preclinical	[16]
IL-2	Increased IL-2 levels post-deprivation.	Preclinical	[36]
IFN-γ	Increased IFN-γ levels post-deprivation.	Preclinical	[36]

## Data Availability

No new data were created in this study. Data sharing is not applicable to this article.
